# 255. Borrelia-specific antibody profiling in joint fluid distinguishes antibiotic-refractory Lyme arthritis from antibiotic-responsive arthritis

**DOI:** 10.1093/ofid/ofad500.327

**Published:** 2023-11-27

**Authors:** Kathryn A Bowman, Christine Wiggins, Elizabeth Deriso, Steffan Paul, Ryan McNamara, Klemen Strle, John A Branda, Allen C Steere, Douglas A Lauffenburger, Galit Alter

**Affiliations:** Ragon Institute of MGH, MIT, and Harvard/Massachusetts General Brigham, Boston, Massachusetts; Massachusetts Institute of Technology, Cambridge, Massachusetts; Ragon Institute of MGH, MIT, and Harvard, Boston, Massachusetts; Harvard Medical School, Boston, Massachusetts; Ragon Institute of MGH, MIT, and Harvard, Boston, Massachusetts; Tufts University School of Medicine, Medford, Massachusetts; Harvard Medical School, Boston, Massachusetts; Massachusetts General Hospital, Boston, Massachusetts; Massachusetts Institute of Technology, Cambridge, Massachusetts; Ragon Institute of MGH, MIT, and Harvard, Boston, Massachusetts

## Abstract

**Background:**

Lyme disease, caused by the spirochete *Borrelia burgdorferi*, is the most common vector-borne disease in the United States. It drives a multisystem disorder, of which Lyme arthritis is the most common feature of late disseminated disease, seen in approximately 60% of untreated individuals. While most Lyme arthritis resolves with oral or IV antibiotics, termed “antibiotic-responsive” arthritis, a small percentage of individuals develop progressive synovitis despite both oral and IV antibiotic therapy, called “antibiotic refractory” Lyme arthritis (LA), requiring treatment with immunosuppressive, disease modifying antirheumatic drugs (DMARDs). The primary drivers behind antibiotic refractory disease are likely multifactorial and remain incompletely understood. More specifically, it remains unclear whether antibodies, specific for *Borrelia* or autoantibodies, may act as biomarkers or play a mechanistic role in driving pathogenesis in the joint.

**Methods:**

We performed a matched, cross-compartmental comparison of antibody profiles from blood and joint fluid of individuals with antibiotic responsive (n = 11) or antibiotic refractory arthritis (n= 31). We used a multiplexed luminex assay to perform biophysical profiling of *Borrelia burgdorferi* -specific antibodies to B67 lysate, CRASP1, CRASP2, DbpA, DbpB, Arp37, Flagellin, OspA, OspB, OspC, OspE, p27, p35, p39, VlsE, and autoantigen Apolipoprotein B100.

**Results:**

While serum antibody profiles poorly discriminated responsive from refractory LA patients, a discrete profile of *B.burgdorferi*-specific antibodies in the joint fluid was able to discriminate antibiotic responsive from refractory LA patients. Cross compartmental comparison of antibody glycosylation, IgA1, and antibody-dependent complement deposition (ADCD) revealed differences across compartments, with more poorly coordinated Lyme-specific humoral responses and increased antibody-dependent complement deposition in antibiotic-refractory arthritis.

**Conclusion:**

These data demonstrate *B.burgdorferi*-specific serological markers that may support the early stratification and clinical management of LA, but also point to immune complex driven complement activation as a key mechanism underlying persistent Lyme arthritis.

**Disclosures:**

**Christine Wiggins, B.S.**, Takeda Pharmaceutical Company: Stocks/Bonds **Elizabeth Deriso, PhD**, Takeda: Employee **Klemen Strle, PhD**, Takeda: I am an employee of Takeda. My work at Takeda does not overlap with my academic efforts in Lyme disease. **John A. Branda, M.D**, Analog Devices Inc.: Grant/Research Support|DiaSorin: Grant/Research Support|Gold Standard Diagnostics: Grant/Research Support|Pfizer Inc: Grant/Research Support|Zeus Scientific: Grant/Research Support **Douglas A. Lauffenburger, PhD**, Sanofi: Board Member|Sanofi: Honoraria|Sanofi: Honoraria **Galit Alter, PhD**, Leyden Labs: Ownership Interest|Moderna Therapeutics: Employee|Seromyx Systems: Ownership Interest

